# Does Sufficient Evidence Exist to Support a Causal Association between Vitamin D Status and Cardiovascular Disease Risk? An Assessment Using Hill’s Criteria for Causality

**DOI:** 10.3390/nu6093403

**Published:** 2014-09-02

**Authors:** Patricia G. Weyland, William B. Grant, Jill Howie-Esquivel

**Affiliations:** 1 Department of Physiological Nursing, School of Nursing, University of California, San Francisco (UCSF), #2 Koret Way Box 0610, San Francisco, CA 94143, USA; E-Mail: jill.howie-esquivel@nursing.ucsf.edu; 2 Sunlight, Nutrition, and Health Research Center, P.O. Box 641603, San Francisco, CA 94164-1603, USA; E-Mail: wbgrant@infionline.net

**Keywords:** association, cardiovascular disease, causation, Hill criteria, vitamin D

## Abstract

Serum 25-hydroxyvitamin D (25(OH)D) levels have been found to be inversely associated with both prevalent and incident cardiovascular disease (CVD) risk factors; dyslipidemia, hypertension and diabetes mellitus. This review looks for evidence of a causal association between low 25(OH)D levels and increased CVD risk. We evaluated journal articles in light of Hill’s criteria for causality in a biological system. The results of our assessment are as follows. Strength of association: many randomized controlled trials (RCTs), prospective and cross-sectional studies found statistically significant inverse associations between 25(OH)D levels and CVD risk factors. Consistency of observed association: most studies found statistically significant inverse associations between 25(OH)D levels and CVD risk factors in various populations, locations and circumstances. Temporality of association: many RCTs and prospective studies found statistically significant inverse associations between 25(OH)D levels and CVD risk factors. Biological gradient (dose-response curve): most studies assessing 25(OH)D levels and CVD risk found an inverse association exhibiting a linear biological gradient. Plausibility of biology: several plausible cellular-level causative mechanisms and biological pathways may lead from a low 25(OH)D level to increased risk for CVD with mediators, such as dyslipidemia, hypertension and diabetes mellitus. Experimental evidence: some well-designed RCTs found increased CVD risk factors with decreasing 25(OH)D levels. Analogy: the association between serum 25(OH)D levels and CVD risk is analogous to that between 25(OH)D levels and the risk of overall cancer, periodontal disease, multiple sclerosis and breast cancer. Conclusion: all relevant Hill criteria for a causal association in a biological system are satisfied to indicate a low 25(OH)D level as a CVD risk factor.

## 1. Introduction

Cardiovascular disease (CVD) is the leading cause of death in the United States and has been since the early 1900s [1]. CVD incidence peaked in the 1960s and then gradually declined over the next 50 years. From 1980 to 2000, the death rate for coronary heart disease (CHD) for men, adjusted for age, decreased from 543 to 267 per 100,000, and for women, the death rate decreased from 263 to 134 per 100,000. Almost half of the decline can be attributed to decreasing CVD risk factors, including hypertension (HTN), smoking and dyslipidemia [2]. The CVD death rate has now plateaued, but, alarmingly, may be increasing [1], reducing life expectancy for the first time [3]. To decrease CVD morbidity and mortality, we must identify and effectively treat all risk factors and their causes.

Robert Scragg [4] first hypothesized that increasing ultra-violet (UV)-related vitamin D status affords protection against CVD. The serum 25-hydroxyvitamin D (25(OH)D) level is the most widely used measurement to assess overall vitamin D status [5]. Serum 25(OH)D levels are inversely associated with several CVDs, including myocardial infarction (MI) [6,7], coronary artery disease (CAD), heart failure, atrial fibrillation, ventricular tachycardia [8], peripheral vascular disease (PVD) [8,9,10,11], stroke [8,12], incident coronary artery calcium (CAC) [13,14,15,16], cardiac valve and vascular calcification [17] and all CVDs [18].

Study findings have inversely associated risk factors for CVD with serum 25(OH)D levels, including lower serum high-density lipoprotein cholesterol (HDL-C) levels, higher serum triglyceride (TG) levels [15], diabetes mellitus (DM) [8,19], increased blood pressure (BP) [15,20,21,22,23,24,25], dysfunctional changes in the characteristics of plasma lipids [26,27,28], inflammation [29] and increased serum parathyroid hormone (PTH) levels [30].

Isolating primary risk factors that cause CVD is challenging, because the human body responds to disrupted homeostasis by up- and down-regulating cellular function. Multiple pathways may exist between a low serum 25(OH)D level and increased CVD risk. Some pathways may be direct and not include any intermediate factors, whereas others may be indirect and include an intermediate factor(s). Moreover, CVD is not a single diagnosis, but rather, according to the National Center for Health Statistics, a group of diagnoses, including CAD, heart failure, essential HTN, hypertensive renal disease, cardiac dysrhythmias, rheumatic heart disease, cardiomyopathy, pulmonary heart disease and cerebrovascular disease [31].

The level of sufficiency for serum 25(OH)D is still being debated. Two schools of thought exist regarding what constitutes a sufficient level: 20 ng/mL [32,33] and 30 ng/mL [34,35,36,37]. Approximately 32% of the U.S. population has a deficient serum 25(OH)D level (defined as <20 ng/mL) [38]. The worldwide prevalence of deficient serum 25(OH)D levels is approximately one billion [39]. The primary causes of low serum 25(OH)D levels are strict sun protection and inadequate dietary or supplemental vitamin D intake [40]. Levels are easily elevated by oral vitamin D supplementation [41]. A daily intake of 10,000–20,000 IU of cholecalciferol (vitamin D_3_) per day is unlikely to result in vitamin D toxicity [42]. Results from epidemiological studies suggest that if a low serum 25(OH)D level is a primary risk factor for CVD and then corrected, all-cause mortality could decrease significantly, both in the United States [43] and worldwide [44].

## 2. Approach and Rationale

The research studies used for this evaluation were located in the PubMed database by using the following search terms: Hill’s criteria for causality, vitamin D, cardiovascular disease, randomized controlled trial, seasonality, hypertension, dyslipidemia, coronary artery calcium, parathyroid hormone, inflammation, diabetes mellitus and high-density lipoprotein cholesterol. Studies were also sought in the references of the preceding studies. We evaluated studies for relevance to this assessment and being representative of current research. We included them regardless of whether they supported criteria for a causal association between serum 25(OH)D levels and CVD risk.

We evaluated the likelihood of a causal association between a low serum 25(OH)D level and increased risk for CVD by applying Sir Austin Bradford Hill’s criteria for causality in a biological system [45] (see Table 1). Causality is multifaceted, and certain conditions must be met to determine that a causal association is likely. Hill stated that the criteria are useful, as we most often depend on observed events to detect relationships between sickness and its antecedents. Waiting to take action until research results explain the entire chain of events that lead to disease may not be necessary when discovering a few links in the chain may suffice.

**Table 1:** Hill’s criteria for causality in a biological system.

Criterion	Defining Question
Strength of the association	Is there a large difference in the outcome between exposed and non-exposed persons?
Consistency of the observed association	Has the outcome been observed by multiple researchers, in various circumstances, places and at different times?
Specificity of the association	Are there specific persons or geographic locations associated with specific outcomes?
Temporality (temporal relationship of the association)	Does the cause always precede the effect?
Biological gradient	Is there a dose-response curve?
Plausibility of the biology	Is the suspected causation consistent with current knowledge of biology?
Coherence	Are there any serious conflicts with the biology or natural history of the disease?
Experiment (experimental or quasi-experimental evidence)	Has an observed association led to a preventive action that has prevented the outcome?
Analogy	Is there an analogous exposure and outcome?

The criteria relevant to this evaluation include all, except specificity and coherence. This evaluation does not include specificity, because evidence supports low serum 25(OH)D levels and increased risk of several other disease processes [43]. This evaluation does not include coherence, because of its similarity to plausibility (see Table 2), and the information would be redundant. Hill’s criteria have been used to assess a causal association between serum 25(OH)D levels and cancer risk [46], periodontal disease [37], multiple sclerosis (MS) [47], breast cancer risk [48] and the most prevalent cancers [49].

To arrive at the most accurate conclusions and to intervene with the most effective treatments, a thorough understanding of causality and of the limitations inherent in how we determine whether a causal association exists is essential. No single type of study, including randomized controlled trials (RCTs), can evaluate each of Hill’s criteria. This evaluation used Hill’s criteria, because it can consider the results of RCTs, prospective, cross-sectional and epidemiological studies.

**Table 2:** Studies used to evaluate causality between low vitamin D and increased risk of CVD. HTN, hypertension; DM, diabetes mellitus; PWV, pulse wave velocity.

Criterion	Proposed Mechanism	Reference	No Effect	Satisfied?
Strength of association		[6,8,12,50,51,52,53]		Yes
Consistency		[7,15,54,55,56]		Yes
Temporality		[8,18,55,57,58]		Yes
Biological Gradient		[8,55,59,60]		Yes
Plausibility	Blunts renin-angiotensin system	[61,62]		Yes
	Arterial stiffness (HTN)	[15,62,63,64,65,66]		
	Reduced risk of DM	[19]		
	Insulin resistance	[67]		
	Glucose regulation	[58,67,68]		
	Seasonal variations in serum 25(OH)D	[4]		
	Lipids	[69,70]		
	Metabolic syndrome	[71,72,73,74,75]		
	DM type 2 and its progression	[19,57,76]		
Experiment	RCTs	[77]	[78]	Yes
	Blood pressure reduction	[79]		
	Blunts renin-angiotensin system	[61]		
	Arterial stiffness (PWV)	[25]		
	Insulin resistance	[80,81]		
	Glucose	[80,81]		
	Lipids		[82,83,84]	
	Metabolic syndrome	[85,86]		
Analogy	Cancer	[46,87]		Yes
	DM type 2	[19]		
Confounding Factors	Nitric oxide liberated by solar UV	[88,89,90]		Yes
	Calcium supplementation	[91]		
	Reverse causation	[91]		
	CVD risk factors affect 25(OH)D levels (obesity)	[91]		
	Physical activity	[92]		
	Statins	[75,93]		
	Seasonal variations in temperature	[94,95]		
Concerns				
Excess vitamin D		[96]		
Hypercalcemia		[97]		
DM	Limited effect of vitamin D	[98,99,101]		

## 3. Findings: Evaluation Using Hill’s Criteria for Causality

The studies included in this criteria section and all of the studies in the subsequent criteria sections are ordered by design; first are the meta-analyses, then prospective, retrospective, cross-sectional, case-control and lastly ecological studies. They are then ordered from the highest to the lowest relative risk ration (RR), hazards ratio (HR) or odds ratio (OR) when available.

### 3.1. Strength of the Association

The stronger the positive or negative association between two variables, the more likely the association is causal. However, this may not always be true. One must consider all that is known about the two variables before concluding that an association is causal. For example, a very strong association may exist between an exposure and a disease, but another unknown variable may mediate the two. Alternatively, an exposure may directly cause a disease, but only under certain, sometimes very limited, circumstances; therefore, the association between the exposure and the disease would be weak. Therefore, a strong association is neither necessary nor sufficient to determine the likelihood of a causal association.

Satisfying the strength of association criterion requires a thorough evaluation of the correlation between vitamin D status and CVD risk. To come as close as possible to determining the true strength of an association, one must determine and then consistently use the most accurate and precise measures of the exposure and the disease [102]. Most researchers agree that the serum 25(OH)D level is the most accurate measure of overall vitamin D status. Several investigators have found statistically significant associations between serum 25(OH)D levels and CVD risk factors or CVDs.

Correia and colleagues [51] performed a prospective study in which they examined the association between serum 25(OH)D levels and the incidence of CVD-related mortalities during hospitalization. Ten percent of their 206 participants were severely deficient, defined as serum 25(OH)D levels ≤10 ng/mL. Incident CVD-related mortality was much higher at 24% for the group of patients with severe serum 25(OH)D deficiency* versus* 4.9% in the group of patients with levels >10 ng/mL (RR 4.3, 95% CI, 1.8, 10, *p* = 0.001). These results are impressive, but the authors acknowledge that the CIs were very wide. Anderson and colleagues [8] completed a study with both cross-sectional and prospective data, which offered support for an association between serum 25(OH)D levels and CVD risk. The researchers examined 41,504 electronic health records and concluded from the cross-sectional data that there is an inverse association between prevalence of CVD risk factors and serum 25(OH)D levels. A significant increase in the prevalence of HTN (30% relative increase RI), DM (90% RI), PVD (53% RI) and hyperlipidemia (9% RI) was present in the group with serum 25(OH)D levels ≤15 ng/mL compared with the group with levels ˃30 ng/mL (*p* < 0.0001 for all, significant after Bonferroni correction for multiple comparisons). The authors acknowledge that selection bias may have been present, because only individuals who had serum 25(OH)D levels in their record were included in the study.

Researchers outside North America have also found inverse associations between serum 25(OH)D levels and risk factors for CVD, although sun exposure and diet may differ. Jang and colleagues [50] performed a cross-sectional study with 320 Korean girls whose average age was 13 years, 63.8% of whom had serum 25(OH)D levels <20 ng/mL. After adjusting for physical activity and BMI *Z*-score, the researchers found that serum 25(OH)D levels were negatively associated with fasting blood glucose levels (*r* = −0.1748, *p* = 0.0033) and insulin resistance (*r* = −0.1441, *p* = 0.0154), both risk factors for metabolic disorders.

The 2013 study by Deleskog and colleagues [53] had mixed results. The researchers performed a cross-sectional study with 3430 participants, 8% of whom had deficient serum 25(OH)D levels defined as <51 nmol/L (<20 ng/mL), 82% had insufficient levels defined as 51–75 nmol/L (20–30 ng/mL) and 10% had sufficient levels defined as ˃75 nmol/L (˃30 ng/mL). No independent association emerged between serum 25(OH)D level insufficiency and carotid intima media thickness. However, those with deficient levels were more likely to have CVD risk factors, including higher BP, blood glucose, TG levels and lower serum HDL-C levels. Additionally, they were more likely to have DM.

Sun and colleagues [12] performed a case-control study in which they examined the association between ischemic stroke risk and serum 25(OH)D levels in 464 females with ischemic stroke and 464 female matched controls. The researchers compared participants in the lowest* versus* highest tertiles of serum 25(OH)D levels after adjusting for dietary and lifestyle covariates. Lower serum 25(OH)D levels were associated with an increased risk for ischemic stroke (OR 1.49, 95% CI, 1.01, 2.18, *p* < 0.04).

Scragg and colleagues [6] were one of the first research teams to examine the association between serum 25(OH)D levels and CVD. The researchers performed a case-control study with 179 MI cases with controls matched for age, sex and date of blood collection. They found an RR for MI of 0.43 (95% CI, 0.27, 0.69) for participants with serum 25(OH)D levels at or above their study median value of 32 nmol/L (12.8 ng/mL)* versus* below the median.

Deleskog and colleagues [52] included 774 participants in a case-control study to evaluate the association between serum 25(OH)D levels and premature MI (younger than 60 years). Serum 25(OH)D levels were analyzed twice as a categorical variable; insufficiency was defined as <50 nmol/L (20 ng/mL) and was compared with levels ≥50 nmol/L; a separate analysis defined insufficiency as <75 nmol/L (30 ng/mL), which was compared with levels ≥75 nmol/L. Neither of the definitions of serum 25(OH)D level insufficiency were independently associated with premature MI. Therefore, the results do not support the criterion. The researchers concluded that the serum 25(OH)D level insufficiency may promote risk factors that are already established and known to promote atherothrombosis.

The criterion strength of the association has thus been met for 25(OH)D levels and CVD or CVD risk factors, including MI, CVD-related mortality, ischemic stroke risk, HTN, DM, PVD, hyperlipidemia, elevated blood glucose and increased insulin resistance.

### 3.2. Consistency of the Association

An association is consistent if it is observed under different circumstances, at different times, in various places and by various researchers [45]. Consistency is also confirmed if the results of a study can be replicated with a different sample of participants with the same study design and analytic methods. Inconsistent study results may occur when differences exist in study design, lab assays, definitions of serum 25(OH)D level deficiency, insufficiency* versus* sufficiency and statistical methods. Confidence in the results of meta-analyses depends on an assessment of the comparability of all studies included in the analysis [103].

Parker and colleagues [54] carried out the study with the strongest support for the criterion of consistency. In their meta-analysis, they systematically reviewed 28 studies with a total of 99,745 participants. The researchers reported important variations among studies included in their review, including categories of serum 25(OH)D levels, study design and analyses. Despite these differences, 29 of 33 ORs from the 28 studies showed an inverse association between serum 25(OH)D levels and the prevalence of cardio-metabolic disorders. One study demonstrated no effect, and three studies showed a positive association. Parker and colleagues [54] found a 43% reduction in cardio-metabolic disorders with the highest levels of serum 25(OH)D (OR 0.57, 95% CI, 0.48, 0.68).

The meta-analysis by Wang and colleagues [55] offers additional strong support. They included 19 prospective studies with a total of 65,994 participants, of whom 6123 developed CVD. The 19 studies included CVD, CVD mortality, CHD and stroke as outcomes. Wang and colleagues found an inverse linear association between serum 25(OH)D in the range 20–60 nmol/L (8–24 ng/mL) and the risk of CVD (RR, 1.03, 95% CI, 1.00, 1.06).

Giovannucci and colleagues [7] found results consistent with the previous studies. This prospective, nested, case-control study included 454 male participants who were CHD cases and 900 male controls matched for age, HTN, aspirin use, physical activity, serum TG and low-density lipoprotein cholesterol (LDL-C) levels, as well as alcohol use. The median values for each of the four categories of serum 25(OH)D levels were entered as continuous variables in a regression model. The researchers found a two-fold increase in risk for MI if the serum 25(OH)D level was less than 16 ng/mL compared with those with a level of at least 30 ng/mL (RR, 2.42, 95% CI, 1.53, 3.84; *p* ˂ 0.001). They also found a 2.1% decreased risk of MI for every 1 ng/mL increase in serum 25(OH)D levels. Only including males in the study prevents the generalizability of the results to females.

Support for the consistency criterion is also evident in the prospective study by de Boer and colleagues [15] (*N* = 1370). At baseline, 723 (53%) had CAC. Over a three-year period, 135 participants developed CAC. The researchers adjusted for gender, age, ethnicity/race, location, season, activity level, smoking status, body mass index (BMI), DM, BP and serum lipid and C-reactive protein (CRP) levels. They found that serum 25(OH)D levels were inversely associated with incident, but not prevalent, CAC; for every 10 ng/mL decrease in the serum 25(OH)D level, the risk of developing CAC increased by 23% (RR, 1.23, 95% CI, 1.00, 1.52, *p* = 0.049).

Finally, a cross-sectional study by Kendrick and colleagues [56] found similar supporting results by using data from 16,603 participants of the Third National Health and Nutrition Examination Survey (NHANES III). Serum 25(OH)D level deficiency, defined as <20 ng/mL, was associated with a 57% increased odds for prevalent CVD. After adjusting for gender, age, ethnicity/race, season, activity level, smoking status, HTN, DM, BMI, dyslipidemia, chronic kidney disease and vitamin D use, the odds decreased to 20% (OR, 1.20, 95% CI, 1.01, 1.36, *p* = 0.03).

A study-participant characteristic that should be included in the evaluation of the consistency criterion is ethnicity. A prospective study by Michos and colleagues [82] found that serum 25(OH)D levels less than 15 ng/mL were not associated with fatal stroke in blacks, but were associated with fatal stroke in whites. One limitation of this study is that because the median time to fatal stroke was 14.1 years and the serum 25(OH)D levels were only drawn once at baseline, there could have been undetected significant changes in serum 25(OH)D levels during the study. Differences in CHD events, including angina, MI, cardiac arrest or CHD death, by ethnicity were found in a prospective study by Robinson-Cohen and colleagues [66]. The researchers found an association between lower serum 25(OH)D levels and incident CHD events for white or Chinese, but not black or Hispanic participants. The same limitation is present in this study; only a baseline serum 25(OH)D level was drawn, and there was a median follow-up period of 8.5 years.

An unexplained difference by ethnicity was found by Gupta and colleagues [104], who performed a cross-sectional study. The researchers found significant associations between both pre-diabetes and pre-hypertension and gender, age and BMI in Mexican-Americans. However, they did not find an association between either pre-diabetes or pre-hypertension and serum 25(OH)D levels, as has been found for both non-Hispanic whites and non-Hispanic blacks. The authors stated that the reason for these results was unclear.

Results from a study performed by Rezai and colleagues [14] found that there was an association between serum 25(OH)D levels and left ventricular end-diastolic volumes for men of all ethnicities. The results of this cross-sectional study add support to the criterion of consistency, because low 25(OH)D levels showed the same association with poorer CV status for all ethnicities. This may mean that disparities in the prevalence of low vitamin D status among ethnicities may cause the disparities among ethnicities in the prevalence of CVD. Webb and colleagues [12] found that the pulse wave velocity (PWV) was higher in British South Asians of Indian descent than in white Europeans (9.32 m/s *vs.* 8.68 m/s, *p* = 0.001) using a cross-sectional design. They also found that the serum 25(OH)D level was independently associated with PWV, when adjusted for age, mean arterial pressure, sex, glucose, heart rate, vasoactive medications and South Asian ethnicity (*R^2^* = 0.73, *p* = 0.004). The researchers concluded that vitamin D insufficiency may mediate an increase in aortic stiffness without a difference in the risk profile, including vascular disease.

The preceding studies have shown mixed results, and the reasons for the differences are multifaceted. One reason for the disparity in serum 25(OH)D levels among different ethnic groups is that vitamin D production is inversely proportional to skin pigmentation [105]. Skin pigmentation varies among members of the same ethnic group, and designing a study in which skin pigmentation is objectively quantified and included as a variable may help to clarify differences between individuals* versus* groups. Studies that use ethnicity self-reporting or that have the investigator determine the ethnicity of the participants can also decrease the validity of the findings.

The consistency of the association criterion has thus been met due to the research results regarding the systematic review by Parker and colleagues and the smaller described supporting studies. The studies regarding ethnicity have mixed results. Parker and colleagues in their meta-analysis found overall associations between serum 25(OH)D levels and MI, stroke, ischemic heart disease, PVD, DM and metabolic syndrome.

### 3.3. Temporality

Temporality refers to the direction of influence in a sequence of events. An event or phenomenon cannot cause another event or phenomenon if the presumed cause does not precede the presumed effect. Determining whether a potential risk factor precedes a disease process is particularly difficult when the disease is chronic and progresses slowly [45]. Determining the temporal direction of influence of low serum 25(OH)D levels in relation to CVD risk by examining the results of prospective studies or meta-analyses that have included only prospective studies will help determine if the criterion of temporality has been met.

DM is a well-established risk factor for CVD, and the association between serum 25(OH)D levels and DM has been prospectively studied. Song and colleagues [57] included 21 prospective studies with 76,220 participants, 4996 incident type 2 DM cases and serum 25(OH)D in a meta-analysis. The researchers compared the highest to lowest serum 25(OH)D levels using categories and found that the summary RR for type 2 DM was 0.62 (95% CI, 0.54, 0.70). The statistical significance of the inverse association between DM risk and serum 25(OH)D levels remained after controlling for sex, criteria for DM diagnosis, follow-up time, sample size and 25(OH)D assay type. Each 10 nmol/mL (4 ng/mL) increase in the serum 25(OH)D level was associated with a 4% lower risk of type 2 DM (95% CI, 3, 6; *p* linear trend = 0.0001). Therefore, low 25(OH)D levels may be a risk factor for CVD with type 2 DM as the mediator.

Wang and colleagues [55] examined the association between CVD mortality along with CVD risk and serum 25(OH)D levels in a meta-analysis of 19 prospective studies. Collectively, these studies had 65,994 participants, of whom 6123 developed CVD. The researchers used the median serum 25(OH)D levels, or if unavailable, they compared the mean or the midpoint of the upper and lower bounds in each of the 25(OH)D categories from each of the 19 studies to the category of the risk of CVD. Being in the lowest category was associated with a higher risk for all CVDs (pooled RR 1.52, 95% CI, 1.30, 1.77), for CVD mortality (pooled RR 1.42, 95% CI, 1.19, 1.71), for CHD (pooled RR 1.38, 95% CI, 1.21, 1.57) and for stroke (pooled RR 1.64, 95% CI, 1.27, 2.10) than the highest category.

Although the study by Anderson and colleagues [8] was included in both the Song and colleagues and Wang and colleagues meta-analyses, it is a landmark study, and the results are important to cite. This study offers strong support for temporality. The prospective study using electronic health records monitored participants for an average of 1.3 years and a maximum of 9.3 years. The prevalence of serum 25(OH)D levels ≤30 ng/mL was 63.6%. Participants without risk factors for CVD with serum 25(OH)D levels ≤15 ng/mL had a higher risk of incident HTN, dyslipidemia and DM than those with levels ˃30 ng/mL. Adjusted relative rates for death increased by 20% for serum 25(OH)D levels of 16–30 ng/mL and increased by 77% for serum 25(OH)D levels ≤15 ng/mL. The researchers concluded that these data provide support for low serum 25(OH)D level as a primary risk factor for CVD. Schöttker and colleagues [18], in a prospective study with 9578 participants, found an increased risk of cardiovascular mortality associated with decreased serum 25(OH)D levels (hazards ratio (HR) 1.39, 95% CI, 1.02, 1.89).

Tsur and colleagues [58] conducted a prospective cohort study over a two-year period that assessed incident impaired fasting glucose (IFG) and DM type 2 in 117,960 participants. The researchers adjusted for several variables, including sex, age, BMI, serum LDL-C, HDL-C, TG levels, history of HTN, smoking status and CVD. Participants with a serum 25(OH)D level ≤25 nmol/L (10 ng/mL) had an OR for progression from normoglycemia to IFG of 1.13 (95% CI, 1.03, 1.24), from normoglycemia to DM of 1.77 (95% CI, 1.11, 2.83) and from IFG to DM of 1.43 (95% CI, 1.16, 1.76), compared with a serum 25(OH)D level ˃75 nmol/L (30 ng/mL). The researchers concluded that a low serum 25(OH)D level may be an independent risk factor for IFG and DM that can eventually lead to CVD.

The previously described meta-analyses of prospective studies and the additional prospective studies offer evidence that temporality is satisfied, because they all use serum 25(OH)D levels taken at the time of enrollment, which precedes the incident event or death. Furthermore, most reviewed individual prospective studies, and a meta-analysis of prospective studies showed an increased incidence of CVD or CVD risk factors with decreasing serum 25(OH)D. The CVDs or risk factors for CVD included CVD mortality, CHD, stroke, dyslipidemia, HTN, type 2 DM and IFG.

### 3.4. Biological Gradient (Dose-Response Relation)

In the context of this assessment, the biological gradient, or dose-response relation, refers to the change in the prevalence or incidence rate of CVD or risk factors for CVD as serum 25(OH)D levels change. The biological gradient criterion is satisfied when the value of the dependent variable (effect) can be predicted, with some degree of confidence, when the value of the independent variable (cause) is known. Hill [45] states that securing a satisfactory quantitative measure to use for this purpose is often difficult.

Wang and colleagues [55] showed a biological gradient effect in their 2012 meta-analysis. They found a linear (graded) and inverse association between serum 25(OH)D levels of 20–60 nmol/L (8–24 ng/mL) and the risk of CVD. They found a linear trend for the RR = 1.03 (95% CI, 1.00, 1.06) for every 25 nmol/L (10 ng/mL) decrease in 25(OH)D ([55]; Figure 3). Wang and colleagues had similar results in an earlier study [60]. They examined low serum 25(OH)D levels and incident CVD prospectively in 1739 participants from the Framingham Offspring Study. A serum 25(OH)D level <15 ng/mL was associated with a two-fold increase in an age and sex-adjusted five-year incident rate for CVD compared with those with a level of ≥15 ng/mL (multivariable-adjusted HR = 1.62, 95% CI, 1.11, 2.36; *p* = 0.01). The researchers also found a graded increase in CVD risk for serum 25(OH)D levels of 10–14 ng/mL (multivariable-adjusted HR = 1.53, 95% CI, 1.00, 2.36; *p* = 0.01)* versus* levels <10 ng/mL (multivariable-adjusted HR = 1.80, 95% CI, 1.05, 3.08; *p* = 0.01).

Anderson and colleagues [8] performed a prospective study, which was included in the Wang and colleagues meta-analysis. The researchers found statistically significant and biologically-graded inverse associations between serum 25(OH)D levels and the prevalence of CVD and CVD risk factors, including PVD, HTN, DM and hyperlipidemia (all *p* < 0.0001). The researchers categorized serum 25(OH)D levels; levels of serum 25(OH)D ≤15 ng/mL* versus* those ˃30 ng/mL were associated with increased prevalence of DM (90% relative and 14% absolute) and HTN (30% relative and 12% absolute) (*p* trend for both <0.0001).

Vacek and colleagues [59] performed a retrospective study (*n* = 10,899) for a 68-month period. Using univariate analysis, the researchers found statistically significant ORs for vitamin D deficiency, defined as ˂30 ng/mL, and CAD (OR, 1.16, 95% CI, 1.012, 1.334, *p* = 0.03), cardiomyopathy (OR, 1.29, 95% CI, 1.019, 1.633, *p* = 0.03) and HTN (OR, 1.40, 95% CI, 1.285, 1.536, *p* ≤ 0.0001).

The criterion, biological gradient, or dose-response curve, has thus been met. Most reviewed studies used serum 25(OH)D levels as categorical or continuous variables and found strong evidence for a graded association between levels and CVD/CVD risk factors, including nonspecific CVD, PVD, HTN, DM, hyperlipidemia, elevated BMI, elevated serum LDL-C and TG levels and decreased serum HDL-C levels.

### 3.5. Plausibility

Biological plausibility can be confirmed when the suspected causation mechanism is consistent with the current knowledge of biology. The actual physiological pathway of the hypothesized causal association between low serum 25(OH)D levels and increased risk for CVD may include mediators that are known CVD risk factors or other unknown factors. Specific cellular-level causative mechanisms that explain the increase in CVD associated with low vitamin D status need to be identified in order to definitively state that the criterion, biological plausibility, has been met.

Several cellular-level causative mechanisms have been proposed. It should be taken into consideration that, in contrast to the causative agent of an infectious disease, these proposed causative mechanisms do not necessarily compete with one another and are not mutually exclusive. Some or all of the proposed mechanisms may be accurate. This is because CVD is a broad category of diseases, and each of the diseases has multiple causes.

An* in vitro* study by Oh and colleagues [17] found an inhibition of foam cell formation when macrophages from persons with type 2 DM exposed to modified LDL were cultured in the bio-active form of vitamin D; 1α,25-dihydroxyvitamin D_3 _(1α,25(OH)_2_D_3_). They also found accelerated foam cell formation when the vitamin D receptors (VDRs) were deleted from the macrophages. A reduction in the formation of atherosclerotic lesions in mice with the administration of the vitamin D analog, calcitriol (1α,25(OH)_2_D_3_), was seen by Takeda and colleagues [32]. They hypothesize that calcitriol modulates the systemic and intestinal immune systems by inducing immunologically-tolerant dendritic cells and T-cells, both of which are anti-atherogenic.

Additionally, an* in vitro* study by Riek and colleagues [106] was performed in order to determine if vitamin D plays a role in monocyte migration and adhesion. The researchers examined monocytes from study participants (*n* = 12) with type 2 DM and obesity who were vitamin D deficient. The researchers found a 20% reduction in monocyte migration in monocytes incubated with 25(OH)D_3_ compared to vitamin D-deficient conditions (*p* < 0.005). They also found that, compared to monocytes maintained in vitamin D-deficient conditions, incubation with 25(OH)D_3_ also significantly decreased adhesion (*p* < 0.05). The researchers concluded that hydroxylation of 25(OH)D_3_ to 1,25(OH)_2_D_3_ at the cellular level may play a role in vitamin D anti-atherogenic effects.

VDRs were also found in human coronary artery smooth muscle cells (CASMC) by Wu-Wong and colleagues [107]. When CASMC were treated with the vitamin D analogs, calcitriol or paricalcitol (19-nor-1α,25(OH)_2_D_2_) there was an upregulation of 24-hydroxylase and also an upregulation of thrombomodulin (TM) mRNA. Downregulation of TM mRNA has been associated with atherosclerosis and thrombosis. Finding that upregulation occurred led the researchers to hypothesize that this is the mechanism that leads to a decrease in morbidity and mortality with vitamin D analog use in persons with chronic kidney disease.

Many studies have shown inverse associations between established CVD risk factors, such as dyslipidemia, HTN and DM [108] and serum 25(OH)D levels (see Table 2). The following research studies further assist in evaluating the plausibility of a causal association.

#### 3.5.1. Dyslipidemia

A proposed causal mechanism for the association between low serum 25(OH)D levels and increased risk for CVD involves dysfunctional changes in the characteristics of plasma lipids, including metabolism or transport [26], the ability to promote macrophage efflux, [27] and changes in serum levels of total cholesterol (total-C), HDL-C, LDL-C and TGs [15,28].

Skaaby and colleagues [70] investigated the association between serum 25(OH)D levels at baseline and incident dyslipidemia over five years in a prospective study with 4330 participants. A serum 25(OH)D level of 10 nmol/L (4 ng/mL) higher at baseline was associated with decreased serum TG levels (β = −0.52, 95% CI, −0.99, −0.05, *p* = 0.03) and decreased serum very-low-density lipoprotein cholesterol (VLDL-C) levels (β = −0.66, 95% CI, −1.1, −0.2, *p* = 0.005). With the same higher serum 25(OH)D level at baseline, the OR for incident hypercholesterolemia was 0.94 (95% CI, 0.90, 0.99, *p* = 0.01). The researchers concluded that higher serum 25(OH)D levels may favorably change lipid profiles and therefore positively influence cardiovascular health.

Karhapää and colleagues [109] performed a cross-sectional study in which they examined the relationship between serum 25(OH)D levels and total-C, LDL-C, HDL-C and TG levels in a study that included 909 male participants. The researchers found a significant inverse association between serum 25(OH)D levels and total-C, LDL-C and TG levels (β = −0.15, −0.13 and −0.17, respectively; *p* < 0.001), which supports lower serum 25(OH)D levels leading to a less favorable lipid profile. However, they found no association between serum 25(OH)D and HDL-C levels, which does not support an association between lower serum 25(OH)D levels and a more favorable lipid profile.

Jorde and colleagues [28] also examined the association between serum 25(OH)D levels and serum lipid levels by using both cross-sectional and longitudinal data collected over 14 years. The cross-sectional study included 10,105 participants, and the researchers found that with increasing quartiles of serum 25(OH)D levels, serum HDL-C and LDL-C levels increased and serum TG levels decreased. In the longitudinal study with 2159 participants, the researchers found that increasing quartiles of serum 25(OH)D levels were associated with decreased serum TG levels. These results, except for the increase in serum LDL-C levels, support associating higher serum 25(OH)D levels with a more favorable lipid profile.

Researchers have also conducted genomic and cytochrome P450 enzyme studies to determine mechanisms that cause low serum 25(OH)D levels to lead to dysfunctional changes in lipids. Shirts and colleagues [69], in a cross-sectional study with 1060 participants, investigated the influence of single-nucleotide polymorphisms on serum HDL-C, LDL-C and TG levels for gene-25(OH)D interactions. Participants with deficient levels of serum 25(OH)D were more likely to also have lower serum HDL-C levels (*p* = 0.0003). Chow and colleagues [110] incubated human hepatocytes with 1,25(OH)_2_D_3 _and found a reduction in cholesterol production due to an increase in cytochrome P450 enzyme 7A1 activation of the VDR.

Guasch and colleagues [29] found an association between low plasma 25(OH)D levels and atherogenic dyslipidemia after adjusting for BMI in a cross-sectional study with 316 participants. When the researchers introduced serum-ultrasensitive CRP levels as a covariable, an association was no longer present. They suggested that inflammation may mediate the effect of serum 25(OH)D levels on lipid profiles.

#### 3.5.2. Hypertension

The cause of HTN is usually unknown. Researchers have investigated the association between serum 25(OH)D levels and both prevalent and incident idiopathic HTN and pre-HTN [20,22,23,24,25]. Carrara and colleagues [61] conducted a prospective interventional trial in which they administered 25,000 IU of oral cholecalciferol (vitamin D_3_) weekly over two months to 15 participants with essential HTN. There was neither randomization to different interventions or a placebo group. Because the researchers found reduced aldosterone (*p* < 0.05) and renin plasma levels (*p* < 0.05) after supplementation, they concluded that for persons with essential HTN and a low serum 25(OH)D level, vitamin D supplementation may help decrease BP.

Forman and colleagues [21] performed a prospective study with 1811 participants with measured plasma 25(OH)D levels. The researchers found that incident HTN was greater for participants with a plasma 25(OH)D level of ˂15 ng/mL compared to those with a level ≥30 ng/mL (RR 3.18, 95% CI, 1.39, 7.29). For men only (*n* = 613), the RR for the same comparison was much greater (RR 6.13, 95% CI, 1.0, 37.8) compared to women only (*n* = 1198) (RR 2.67, 95% CI, 1.05, 6.79). Forman and colleagues [62] also conducted a prospective study that included only women participants aged 32–52 years. The researchers found that incident HTN increased for the lowest quartile (6.2–21.0 ng/mL) *versus* the highest quartile (32.3–89.5 ng/mL) for 25(OH)D levels (OR, 1.66, 95% CI, 1.11, 2.48,* p* = 0.01).

Increased arterial stiffness may be an effect of low serum 25(OH)D level. Giallauria and colleagues [63], in a cross-sectional study with 1228 participants, found a statistically significant inverse association between serum 25(OH)D levels and arterial stiffness, measured with PWV (adjusted *R^2^* = 0.27, β = −0.43; *p* = 0.001). Furthermore, measuring PWV, Mayer and colleagues [64] performed a cross-sectional study and found a negative association with serum 25(OH)D level quartiles. The lowest serum 25(OH)D level quartile (<20 ng/mL) had the highest PWV score compared with the second, third or fourth quartile (*p* = 0.0001).

Three studies with only female participants had similar results. Pirro and colleagues conducted a cross-sectional study with 150 postmenopausal and serum 25(OH)D-insufficient (<30 ng/mL) participants [65]. The researchers found a significant association between arterial stiffness, measured with PWV and serum 25(OH)D levels, but not after controlling for logarithmically-transformed serum PTH levels. Serum PTH levels were associated with arterial stiffness (β = 0.23, *p* = 0.007). Reynolds and colleagues [66] in a cross-sectional study found a similar association between serum 25(OH)D levels and aortic stiffness (PWV scores) (β = −0.0217, 95% CI, −0.038, −0.005, *p* = 0.010) for 75 female participants with systemic lupus erythematosus. The authors did not state that serum PTH levels were measured and controlled for, and therefore, PTH levels may have mediated the association.

#### 3.5.3. Diabetes Mellitus

DM is an important risk factor for CVD. Several studies have associated serum 25(OH)D levels and both prevalent and incident DM. Afzal and colleagues [19], in a prospective study with 9841 white participants, found an increased risk of type 2 DM for study participants with plasma 25(OH)D levels ˂5 ng/mL* versus* ≥20 ng/mL (HR, 1.22, 95% CI, 0.85, 1.74). The researchers also performed a meta-analysis of 13 studies and found a greater prevalence of type 2 DM for those in the lowest* versus* highest quartile for the serum 25(OH)D level (cut-points for the quartiles varied among the 13 studies) (OR, 1.39, 95% CI, 1.21, 1.58). Anderson and colleagues [8], found an adjusted RI in incident DM of 89% for very low (≤15 ng/mL)* versus* sufficient (˃30 ng/mL) categories of serum 25(OH)D levels (HR, 1.89, 95% CI, 1.54, 2.33, *p* < 0.0001). Forouhi and colleagues [67] found in a prospective study with 524 participants that baseline 25(OH)D levels were inversely associated with the 10-year risk of hyperglycemia (fasting glucose: β = −0.002, *p* = 0.02) and insulin resistance (fasting insulin β = −0.15, *p* = 0.01).

#### 3.5.4. Metabolic Syndrome

Studies have been conducted to assess the association between both incident and prevalent metabolic syndrome and serum 25(OH)D levels. A prospective study by Gagnon and colleagues [74] found that 12.7% of 4164 participants developed metabolic syndrome over a five-year follow-up period. A higher risk of metabolic syndrome was present for those with serum 25(OH)D levels in the first quintile (<18 ng/mL) (OR = 1.41, 95% CI, 1.02, 1.95) and second quintile (18–23 ng/mL) (OR = 1.74, 95% CI, 1.28, 2.37) compared with the highest quintile (≥34 ng/mL). Serum 25(OH)D levels were inversely associated with fasting glucose (*p* < 0.01), homeostasis model assessment for insulin resistance (*p* < 0.001), TG (*p* < 0.01) and waist circumference (*p* < 0.001). No association with two-hour plasma glucose (*p* = 0.29), HDL-C (*p* = 0.70) or BP (*p* = 0.46) was evident at the five-year follow-up.

Another cross-sectional study conducted by Brenner and colleagues [72] with 1818 participants found an 8.9% prevalence of metabolic syndrome. The researchers found an inverse association between plasma 25(OH)D levels and the number of components for metabolic syndrome (β = −0.1, *p* < 0.0001). Components of metabolic syndrome included serum HDL-C level <40 mg/dL (males) or <50 mg/dL (females), serum TG level ˃1.7 mmol/L, fasting plasma glucose ˃110 mg/dL, BP ˃ 130/85 mmHg and waist circumference >102 cm (males) or >88 cm (females). A lower OR (0.50, 95% CI, 0.24, 1.06) for metabolic syndrome was evident for study participants whose plasma 25(OH)D level was in the highest* versus* lowest quartile. After adjusting for age, sex, ethnicity, smoking status, physical activity and month of interview, researchers found that a 10-nmol/mL (4 ng/mL) increase in the plasma 25(OH)D level was inversely associated with the homeostasis model assessment for insulin resistance score (β = −0.08, *p* = 0.006). Another cross-sectional study by Reis and colleagues [71] that included 1654 participants with DM assessed the prevalence of metabolic syndrome. The researchers divided serum 25(OH)D levels into quintiles and found an OR of 0.27 (CI, 0.15, 0.46; *p* trend <0.001) for metabolic syndrome for the highest quintile (median = 88 nmol/L (35 ng/mL))* versus* the lowest quintile (median = 26.8 nmol/L (10.7 ng/mL)).

In a case-control study by Makariou and colleagues, 52 participants with metabolic syndrome had lower serum 25(OH)D levels (mean = 11.8 ng/mL, range = 0.6–48.3 ng/mL) than 58 controls (mean = 17.2 ng/mL, range = 4.8–62.4 ng/mL; *p* = 0.027) [75]. Serum 25(OH)D levels were inversely associated with serum TG levels (*r* = −0.42, *p* = 0.003) and small dense LDL-C (*r* = −0.31, *p* = 0.004).

The criterion for plausibility has thus been satisfied. There are several proposed biologically-plausible cellular-level mechanisms for the increase in CVD associated with low vitamin D status. Studies involving the assessment of an association between serum 25(OH)D levels and dyslipidemia, HTN, DM and metabolic syndrome have also been evaluated. Dyslipidemia, HTN, DM and metabolic syndrome are all plausible mediators between low serum 25(OH)D levels and increased risk of CVD. Specifically, the studies support increased serum LDL-C, VLDL-C and TG levels, decreased serum HDL-C levels, increased arterial stiffness, increased insulin resistance, hyperglycemia and increased incident metabolic syndrome as potentially plausible mediators.

### 3.6. Experiment

Researchers have conducted RCTs to assess the effect of serum 25(OH)D levels on CVD risk factors. However, vitamin D RCTs conducted to date have mixed results. The main reason is that vitamin D RCTs have been designed largely on the model used for pharmaceutical drugs, which assumes that the agent used in the trial is the only source of the agent and that a linear dose-response relation exists. Neither assumption is valid for vitamin D. 

Another consideration is that chronic disease is caused by more than one risk factor and may occur only after long-term* versus* short-term vitamin deficiency. Vitamin supplementation studies are usually designed to assess the decrease in risk due to increasing vitamin intake to meet the minimum sufficiency level. Additional information would be gained from studies that also test the effects of supplementation on levels beyond those previously established for disease risk [111].

Robert Heaney [112] recently outlined the steps to design and conduct vitamin D RCTs: (1) start with the 25(OH)D level-health outcome relation; (2) measure the 25(OH)D levels of prospective participants; (3) enroll only those with low 25(OH)D levels; (4) supplement with enough vitamin D_3_ to increase 25(OH)D levels to the upper end of the quasi-linear region of the 25(OH)D level-health outcome relation; and (5) re-measure 25(OH)D levels after supplementation. For CVD, these recommendations would translate to enrolling people with 25(OH)D levels below about 15 ng/mL and then supplementing with 2000–4000 IU of vitamin D_3_ per day to raise 25(OH)D levels to >30–40 ng/mL.

The effect of vitamin D supplementation on CVD risk factors for women with polycystic ovarian syndrome was investigated by Rahimi-Ardabili and colleagues [113]. The study participants taking the vitamin D supplement had a statistically significant increase in serum vitamin D level and statistically significant decreases in serum total-C, TGs and VLDL-C levels (all *p* < 0.05). They did not have any changes in serum levels of HDL-C, LDL-C, apolipoprotein-A1 (Apo-A1) or high-sensitivity C-reactive protein (hs-CRP). The placebo group had no changes.

Schnatz and colleagues [77] supplemented participants (*n* = 600) with 1000 mg of elemental calcium and 400 IU of vitamin D per day. The researchers found a 1.28 mg/dL decrease in LDL-C (*p* = 0.04) with a 38% increase in the 25(OH)D level. The researchers also found an increase in HDL-C and a decrease in TGs.

Breslavsky and colleagues [68] conducted an RCT, including 47 participants with type 2 DM, who were randomized into two groups. One group received cholecalciferol (vitamin D_3_) at 1000 IU per day for 12 months, whereas the other group received a placebo. After being similar at baseline, the group receiving cholecalciferol had significantly decreased hemoglobin A1c levels (*p* < 0.0001), but no change occurred in the placebo group.

Grimnes and colleagues [114] performed an RCT with 94 participants with low serum 25(OH)D levels. The participants were randomly assigned to receive a 20,000 IU supplement of oral D_3_ or a placebo twice weekly for six months. The supplement did not improve the lipid profile, which included total-C, LDL-C, HDL-C and TGs.

Ponda and colleagues [82] conducted a randomized, placebo-controlled, double-blinded trial. They randomized 151 vitamin D-deficient participants to receive oral D_3_ at 50,000 IU weekly for eight weeks or placebo and then examined the effect on serum cholesterol levels. In the supplemented group, serum 25(OH)D levels increased, serum PTH levels decreased and serum calcium levels increased. When participants were stratified by the change in serum 25(OH)D level and the serum calcium level, those whose response was greater than the median response had an increase in serum LDL-C of 15.4 mg/dL compared with those who had lower than the median response. The analysis of the group receiving placebo did not show this relationship. Table 3 shows results from RCTs in order of serum 25(OH)D level at time of enrollment. This RCT does not support a beneficial effect on lipid status.

In a manuscript under preparation, it was found that for vitamin D RCTs related to CVD risk factors, the median baseline serum 25(OH)D level for the RCT with significant beneficial effects was 15 ng/mL, while the median baseline serum 25(OH)D for those without beneficial effects was 19 ng/mL (Grant, in preparation). This finding underscores the importance of having a low baseline serum 25(OH)D level when designing and conducting vitamin D RCTs to evaluate the findings of observational studies, as proposed by Heaney [112].

The criterion for experiment has thus been met. We reviewed RCTs that supplemented participants with vitamin D and found that most well-designed RCTs supported a causal association between serum 25(OH)D levels and CVD risk.

**Table 3:** Results of studies on vitamin D supplementation and CVD risk factors (ordered by mean serum 25(OH)D (ng/mL)).

Mean Serum 25(OH)D (ng/mL)	Vitamin D_3_ Dose (IU/day)	Increase in 25(OH)D (ng/mL)	Mean Age (year)	Health Outcome of Interest	Findings	Reference
8.4	4000	19.6	42	Insulin sensitivity	5.9 *vs.* −5.9 (*p* = 0.003)	[115]
8.4	4000	19.6	42	Fasting serum glucose	−3.6 *vs.* 1.1 (*p* = 0.02)	[115]
13	400 or 1000	13	64	HDL-C, LDL-C, TG, ApoA1, ApoB100, HOMA-IR, hs-CRP, sICAM-1, IL-6	Not significant	[83]
<20	2000 *			Total cholesterol, HDL-C, LDL-C, TGs	Not significant	[82]
14.7	1000	15	38	Total cholesterol, LDL-C, ApoA1, ApoA1:ApoB-100	Significant to *p* < 0.01	[108]
14.7	1000	15	38	HDL-C, LDL-C:ApoB-100	Significant to *p* < 0.04	[108]
14.7	1000	15	38	ApoB-100, lipoprotein(a)	Not significant	[108]
16.1	2857 *	40	52	Insulin sensitivity	Not significant	[114]
16.3	0		51	Systolic BP	+1.7 mm	[79]
16.3	1000		51		−0.66 mm	[79]
14.5	2000		51		−3.4 mm	[79]
15.6	4000		51		−4.0 mm	[79]
19.6	4000	19.5	14.1	Insulin sensitivity	−1.36 *vs.* +0.27 (*p* = 0.03)	[116]
				Fasting insulin	−6.5 *vs.* +1.2 (*p* = 0.03)	[116]
19.6	2857 * or 5714 *	40	52	HDL-C, LDL-C, TGs, ApoA1, ApoB, hs-CRP	Not significant	[84]
22.9	2857 * or 5714 *	22.8	50	TNF-α, IL-6, HOMA-IR, QUICKI	Not significant	[117]
23	1000	21	61	Systolic BP	−1.5 mm* vs.* +0.4 mm (*p* = 0.26)	[25]
30.3	2500	16	64	Glucose, CRP, FMD, diastolic BP, systolic BP, PWV	Not significant	[118]

* Average daily oral intake from a bolus dose; FMD, flow-mediated dilation; QUICKI, qualitative insulin sensitivity check index; hs-CRP, high-sensitivity C-reactive protein; TNF-α, tissue necrosis factorα; IL-6, interleukin 6; ApoA1, apolipoprotein A1; ApoB, apolipoprotein B.

### 3.7. Analogy

The likelihood of a causal association between low vitamin D status and several diseases has been evaluated using Hill’s criteria for causality in a biological system. Hill’s criteria were met when Grant [46] evaluated overall cancer risk, when breast cancer risk was evaluated by Mohr and colleagues [48], when Grant and Boucher [37] evaluated periodontal disease and when Hanwell and Banwell [47] evaluated multiple sclerosis. Hanwell and Banwell found that all of the criteria were satisfied, except the criterion for disease prevention by intervention (experiment). The researchers state that fulfilling this criterion will be difficult because multiple sclerosis has a low incidence, the age of onset is highly variable and there is a lack of consensus regarding optimal vitamin D dose and the timing of treatment.

The criterion, analogy, has thus been met. Several assessments with various diseases have shown an analogous association to low serum 25(OH)D levels and CVD risk.

### 3.8. Confounding Factors

Potischman and colleagues [119] discussed the inadequacies of traditional causal criteria for assessing nutrients, but they acknowledged that they are necessary for public health recommendations. The authors stated that additional important considerations exist, such as confounding, errors in measurement and dose-response curves for nutrients.

Opländer and colleagues [88] discovered a potentially confounding factor for the association between production of vitamin D in the skin and a decrease in BP. UVB irradiation is responsible for vitamin D production and is associated with a decrease in BP, but UVA irradiation was found to also decrease BP. The effect was attributed to UVA irradiation-induced release of nitric oxide.

Beveridge and colleagues identified other confounders [91]. Associations in vitamin D studies may be confounded by the effects of other CVD risk factors in addition to those being studied, and confounding related to the possibility of reverse causality may also occur. Liberopoulos and colleagues [93] found that statins have different effects on the increase of serum 25(OH)D levels. Woodhouse and colleagues [94] found a seasonal variation in serum total-C, HDL-C and TG levels. These confounders can be controlled for with the use of appropriate statistical analyses, just as age, gender, ethnicity, BMI and smoking status are often controlled for in research studies.

Essential to the credibility of study results is the measurement and reporting of adherence to the intervention. The evaluation of adherence to oral vitamin D supplements given in a study may be either absent or inadequate. Furthermore, an inquiry about concurrent use of personal oral vitamin D supplementation may differ across studies. Negative study results may simply be attributed to a lack of adherence to the intervention, because it leads to bias and a decrease in the statistical power. 

## 4. Conclusions

Despite the identification and treatment of currently recognized CVD risks, CVD remains the leading cause of death. The focus of vitamin D research has recently expanded to include the effects of vitamin D status on CVD and CVD risk factors. Low serum 25(OH)D levels are associated with increased incidence [8], prevalence [56] and risk factors for CVD [15]. This assessment demonstrates that Hill’s criteria were satisfied.

Potential benefits of decreasing the impact of a risk factor for CVD should outweigh potential risks. Repletion of vitamin D stores with a supplemental dose of 10,000 IU per day or less is unlikely to lead to toxic effects [39]. Repletion can be accomplished by a sensible increase in sun exposure [37] or by consuming vitamin D-rich foods, but this goal is most easily accomplished with oral supplementation. Furthermore, more severe deficiencies in serum 25(OH)D levels show a more rapid increase than less severe deficiencies [103]. Treatment for some CVD risk factors is expensive and may be difficult to access, but oral vitamin D supplements are readily accessible and reasonably priced. Other considerations for individualized treatment should include attention to skin melanin content, latitude and altitude of residence, dietary habits and amount of sun exposure.

The physiological mechanisms hypothesized to cause low vitamin D status to increase CVD risk have not yet been confirmed. Nearly all research studies regarding low vitamin D status and increased risk of CVD use observational study designs. More RCTs are needed that incorporate the complex pharmacokinetic and pharmacodynamic properties of vitamin D in the study design: dose-response curve, half-life, avoidance of toxicity and use of the most accurate and precise serum assays.

Exposure to sunlight or vitamin D supplementation may be used in an RCT, although having a control group with a zero serum 25(OH)D level would not be possible. This approach is possible only in drug studies [120]. Nutrients are more appropriately studied in the context of proving negative causation: the absence of an antecedent caused the consequence. This study design would be consistent with research involving preventive healthcare strategies.

Current scientific evidence supports a causal association between serum 25(OH)D levels and increased risk for CVD on the basis of Hill’s criteria for causality in a biological system. Only RCTs starting with low serum 25(OH)D levels found significant beneficial effects of vitamin D supplementation in reducing risk factors associated with CVD. However, evidence to date suggests that raising serum 25(OH)D levels to at least 30 ng/mL will reduce the risk of CVD.

Whether it is ethical to design a study in which a group of people is deprived of a known essential nutrient to measure an endpoint should be carefully determined. Furthermore, waiting for completion of long-term RCTs to change treatment recommendations, especially when risks are minimal, may adversely affect the health of countless individuals. According to Hill [45]: “All scientific work is incomplete—whether it be observational or experimental. All scientific work is liable to be upset or modified by advancing knowledge. That does not confer upon us a freedom to ignore the knowledge we already have, or to postpone the action that it appears to demand at a given time.”
